# Hemorrhagic cerebral infarction in carbon monoxide poisoning: a case report

**DOI:** 10.1186/1757-1626-2-96

**Published:** 2009-01-29

**Authors:** Mostafa El Khashab, Farideh Nejat

**Affiliations:** 1 Department of Neurosurgery, Hackensack University Medical Center, New Jersey, USA; 2 Department of Neurosurgery, Children's Hospital Medical Center, Tehran University of Medical Sciences, Tehran, Iran

## Abstract

**Introduction:**

Almost every known central neurological syndrome has been reported as a complication of carbon monoxide poisoning. Hemorrhagic infarct has rarely been considered as an early manifestation of carbon monoxide poisoning. A case of cerebral hemorrhagic infarction is presented. Typical findings, neuropathology and the role of vascular injury are described.

**Case presentation:**

The symptoms and clinical course of acute poisoning with carbon monoxide in a 7-year-old boy are described. To evaluate the possible causes, a brain computed tomography (CT) was performed that showed thick clot in the left medial temporal and parasellar area, left sylvian fissure(acute intravascular thrombus) accompanied by left diffuse frontotemporal hypodensity and midline shift. Four-vessel digital subtraction angiography two weeks after intoxication was not indicative of any vascular lesion.

**Conclusion:**

Hemorrhagic infarction is a rare presentation of carbon monoxide poisoning. When found in a child, in addition to conservative treatment to reduce the neurocognitive squeal, other probable causes should be ruled out.

## Introduction

Carbon monoxide (CO) poisoning is a leading cause of poisoning death. Infants and children may be at a higher risk of toxicity because their exposure is increased by high minute ventilation volume and high metabolic rate. Commonly the cerebral lesions occur in the basal ganglia, and central gray and white matters. Pathologic changes can include necrosis of globus pallidus, spongy change in the cerebral cortex, and necrosis or demyelination in the cerebral or cerebellar white matter [1,2]. We describe a temporo-frontal hemorrhagic infarction demonstrated by computerized tomography scan (CT) in a child with CO exposure.

## Case presentation

In the winter, a 7-year-old boy was admitted with history of persistent vomiting, progressive loss of consciousness and sever right hemiparesis. He had been well several hours before admission when he and all of his family members had developed vomiting, headache and lethargy.

Carbon monoxide poisoning was suspected based on a history of CO leakage from a gas stove at night subsequent to chimney obstruction, and similar symptoms in other co-habitants. All of them were admitted to hospital and subsequently had good recovery with standard treatment for CO poisoning. However, this young boy showed progressive worsening of symptoms including persistent vomiting, generalized convulsions, coma, and right hemiparesis.

Blood analysis revealed pH 7.4, PCO_2 _35 mmHg, PO_2 _125 mmHg, a total hemoglobin concentration of 13 g with 75% HbO_2_, 24.5% HbCO and 0.5% metHb.

**Figure 1:**
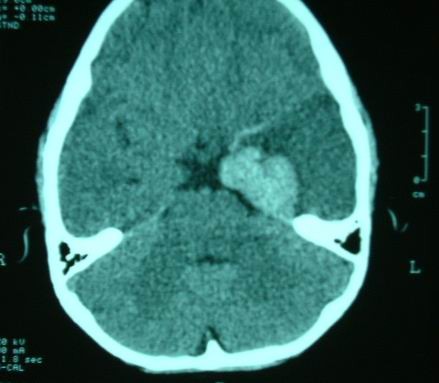
**Brain CT scan of the patient demonstrates thick clot in the left medial temporal and clot in left middle cerebral artery accompanied by left temporal hypodensity**.

**Figure 2:**
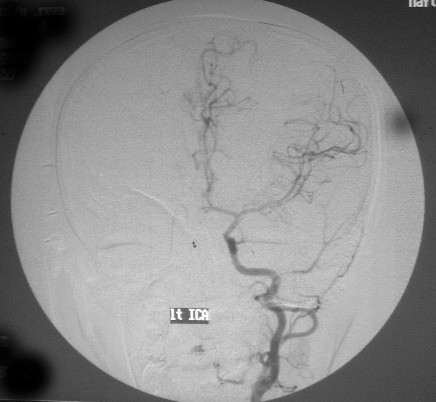
**Left carotid angiogram shows normal anatomy of left carotid and middle cerebral arteries**.

**Figure 3:**
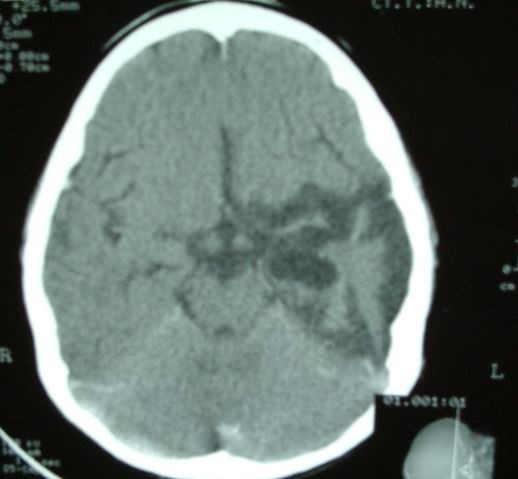
**A, B: Follow-up CT scan demonstrates severe brain atrophy in previous hypodense area which confirms infracted brain was resolved**.

Brain CT was performed on the day after admission which showed thick clot in left medial temporal and parasellar areas, intravascular thrombus in the left middle cerebral artery (Fig 1), diffuse left fronto-temporal hypodensity, and midline shift. Inspite of well known history of CO poisoning and brain CT suggestive of extensive hemorrhagic infarction, DSA angiography was done to exclude gross cerebral abnormalities but it was normal (Fig 2). A good recovery was found except for a global aphasia. Follow-up brain CT scan one year later revealed brain atrophy in left fronto-temporal area, which appeared to have been an evidence of resolving in a large hemorrhagic infarct (Fig 3).

## Discussion

Carbon monoxide poisoning can produce three different forms of symptoms: acute intoxication, recurrent symptom syndrome and delayed neurocognitive or neuropsychiatric squeal. The clinical manifestations are influenced by both the duration of exposure and the concentration of CO in the environment. The toxic effects result predominantly from the interaction of CO with hemoglobin and also with other hem proteins such as myoglobin, cytochrome oxidase, cytochrome P-450, catalases and peroxidases. From anatomical perspective, the most common lesions occur in the basal ganglia and cerebral gray and white matters [1]. Cerebellar white matter damage has been explained. There is also loss of neurons in the cerebral and cerebellar cortices and so in the basal ganglia [1,2]. Pathologically, white matter changes range from small multifocal necrotic areas in the commissural and cerebral centrum to extensive zones of necrosis throughout both hemispheres. Symmetric subcortical demyelination is common and may account for delayed neurological deterioration. Cerebral edema can also be seen in severe poisoning and may lead to brain damage and focal neurological signs [3]. Focal hemorrhage in basal ganglia of patients with CO poisoning has been reported in autopsy and MRI findings [4]. Hemorrhagic infarct has rarely been considered as an early manifestation of CO poisoning [3,4]. The superiority of MRI over CT scan in detecting the presence of extravasated blood and establishing the diagnosis of hemorrhagic brain infarction has been well documented [5]. Brain CT scan is unable to detect petechial hemorrhage in patients with CO poisoning and can only show low-density areas of edema around the petechial hemorrhage [4].

Hemorrhagic infarction in the brain of patients referred for CO poisoning is very rare. We have tried to describe the probable mechanisms. Micro-vascular impairment and brain reperfusion injury patterns have been reported in CO poisoning and appear to be derived from uncontrolled oxidative damage initiated by oxygen free radicals, and sustained by second-generation lipid radicals. In addition, endothelial homeostasic disturbance due to local hypoxic injury from co-mediated mitochondria dysfunction has been documented in CO poisoning. Attraction of polymorph nuclear leukocytes to the site of oxidative endothelial injury perpetuates further free radical mediated injury. This micro vascular injury can produce ischemia and hemorrhage. Besides cardiovascular and hematological complications due to CO poisoning have been proposed to be another cause for hemorrhage.

According to history, initial similar symptoms in all of the family members (without pervious history supporting the present symptoms), and the laboratory test, it is logical to consider the case as a complication of CO poisoning. Brain CT revealed temporal lobe hemorrhage and intravascular thrombus inside left middle cerebral artery. The patient likely had hemorrhagic infarction. Although the hemorrhage was predominantly intracerebral. Digital subtraction angiography was done which couldn't find visible great vessel damage or incidental vascular abnormalities.

## Conclusion

Hemorrhagic infarction is a rare presentation of carbon monoxide poisoning. When found in a child, in addition to conservative treatment to reduce the neurocognitive squeal, other probable causes should be ruled out.

## Consent

Written informed consent was obtained from the patient's parents for publication of this case report. A copy of the written consent is available for review by the Editor-in-Chief of this journal.

## Competing interests

The authors declare that they have no competing interests.

## Authors' contributions

Both authors made contribution to conception, analyzed the patient data, and drafting the manuscript.

## References

[B1] Bianco F Floris R MRI appearances consistent with hemorrhagic infarction as an early manifestation of carbon monoxide poisoning Neuroradiology 1996 38 Suppl 1 S70 2 10.1007/BF02278123 8811684

[B2] Mascalchi M Petruzzi P MRI of cerebellar white matter damage due to carbon monoxide poisoning: case report Neuroradiology 1996 38 S73 74 10.1007/BF02278124 8811685

[B3] Schils F Cabay JE Flandroy P Dondelinger RF Unusual CT and MRI appearance of carbon monoxide poisoning JBR-BTR 1999 82 1 13 5 11155858

[B4] Sawada Y Sakamoyo T Nishide K Sadamitsu D Correlation of pathological findings with CT finding after acute COP N E J Med 1983 308 1296 6843619

[B5] Silverman CS Brenner J Murtagh FR Hemorrhagic necrosis and vascular injury in carbon monoxide poisoning: MR demonstration AJNR Am J Neuroradiol 1993 14 1 168 70 8427081 PMC8334460

